# Supplementary material for: Knowledge about traumatic World War II experiences among ancestors and subjective well-being of young adults: A person-centred perspective
Source: PLoS One. 2020 Aug 24;15(8):e0237859. doi: 10.1371/journal.pone.0237859 (PMC7446788; doi:10.1371/journal.pone.0237859)
Supplement: S1 File — (DOCX) [file pone.0237859.s001.docx]

PAMIĘĆ ZDARZEŃ Z OKRESU II WOJNY ŚWIATOWEJ - KWESTIONARIUSZ

Maja Lis-Turlejska, Anna Ćwiklińska, Piotr Halimoniuk

Ten kwestionariusz został stworzony z myślą o ustaleniu wiedzy na temat zdarzeń traumatycznych podczas II wojny światowej, które przeżyli członkowie Pani/Pana rodziny. W zależności od wieku może on dotyczyć pradziadków, dziadków, babcie lub prababcie.

Poniżej znajdują się dwie tabele. Prosimy o przeczytanie pytań znajdujących się po lewej stronie i o zaznaczenie odpowiedzi znakiem (X) w rubryce TAK, jeśli wie Pani/Pan że takie zdarzenie miało miejsce – pradziadek/prababcia, dziadek/ babcia, mówili o tym lub dowiedziała się Pani/Pan o tym w inny sposób. Jeśli zdarzenie nie miało miejsca , prosimy o zaznaczenie tego w rubryce NIE. Jeśli nic Pani/Panu nie wiadomo na ten temat, prosimy o zaznaczenie tego w rubryce NIE WIEM. Jeśli któreś z pradziadków lub dziadków urodziło się po 1945 roku, prosimy pominąć odpowiednie kolumny. Pierwsza tabela dotyczy przodków ze strony matki. Druga - ze strony ojca.

# (PRA)DZIADKOWIE/(PRA)BABCIE STRONY MATKI:

Rok urodzenia …….............proszę wpisać o kogo chodzi ze strony matki ………………………………..…………..

| **ZDARZENIE** | | **(PRA)BABCIA** | | | **(PRA)DZIADEK** | | |
| --- | --- | --- | --- | --- | --- | --- | --- |
|  |  | **TAK** | **NIE** | **NIE WIEM** | **Tak** | **Nie** | **NIE WIEM** |
| **Czy podczas wojny Pani/Pana (pra)babcia/dziadek:** | | | | | | | |
| 1. | Straciła/stracił matkę? |  |  |  |  |  |  |
| 2. | Straciła/stracił ojca? |  |  |  |  |  |  |
| 3. | Stracił/a inną bliską osobę? |  |  |  |  |  |  |
| 4. | Brał/a udział w walce frontowej? |  |  |  |  |  |  |
| 5. | Był/a w partyzantce? |  |  |  |  |  |  |
| 6. | Był/a ranny/a? |  |  |  |  |  |  |
| 7. | Zabił/a kogoś? |  |  |  |  |  |  |
| 8. | Był/a torturowana? |  |  |  |  |  |  |
| **ZDARZENIE** | | **(PRA)BABCIA** | | | **(PRA)DZIADEK** | | |

|  | | **TAK** | **NIE** | **Nie wiem** | **Tak** | **Nie** | **Nie wiem** |
| --- | --- | --- | --- | --- | --- | --- | --- |
| **Czy podczas wojny Pani/Pana (pra) babcia/dziadek:** | | | | | | | |
| 9. | Był/a w hitlerowskim obozie koncentracyjnym? |  |  |  |  |  |  |
| 10. | Był/a w łagrze? |  |  |  |  |  |  |
| 11. | Był/była w getcie? |  |  |  |  |  |  |
| 12. | Była w Warszawie podczas  Powstania Warszawskiego? |  |  |  |  |  |  |
| 13. | Doświadczył/a gwałtu lub innej  formy przemocy seksualnej? |  |  |  |  |  |  |
| 14. | Przeżył/a bombardowanie? |  |  |  |  |  |  |
| 15. | Musiał/a się ukrywać? |  |  |  |  |  |  |
| 16. | Ukrywał/a Żydów? |  |  |  |  |  |  |
| 17. | Był/a przymusowo wysiedlona na Syberię? |  |  |  |  |  |  |
| 18. | Był/a na robotach przymusowych  na terenie III Rzeszy? |  |  |  |  |  |  |
| 19. | Doświadczył/a zagrażającego życiu  mrozu? |  |  |  |  |  |  |
| 20. | Doświadczył/a zagrażającego życiu głodu? |  |  |  |  |  |  |
| 21. | Walczył/a w partyzantce lub w ruchu oporu (Armia Krajowa, Gwardia Ludowa, innych)? |  |  |  |  |  |  |
| 22. | Był/a poważnie chora? |  |  |  |  |  |  |
| 23. | Przeżył/a fakt, że ktoś z jego/jej najbliższych był poddany  torturom, doznał gwałtu,  przemocy seksualnej lub został  poważnie ranny? |  |  |  |  |  |  |
| **ZDARZENIE** | | **(PRA)BABCIA** | | | **(PRA)DZIADEK** | | |

|  | | **TAK** | **NIE** | **NIE WIEM** | **TAK** | **NIE** | **NIE WIEM** |
| --- | --- | --- | --- | --- | --- | --- | --- |
| **Czy podczas wojny Pani/Pana (pra)babcia/dziadek była/był świadkiem:** | | | | | | | |
| 24. | Walki frontowej? |  |  |  |  |  |  |
| 25. | Postrzelenia kogoś? |  |  |  |  |  |  |
| 26. | Egzekucji, zabicia kogoś? |  |  |  |  |  |  |
| 27. | Gwałtu lub innej formy przemocy  seksualnej? |  |  |  |  |  |  |
| 28. | Ciężkiego pobicia kogoś? |  |  |  |  |  |  |
| 29. | Napaści lub prześladowania Żydów? |  |  |  |  |  |  |

# (PRA)DZIADKOWIE/(PRA)BABCIE STRONY OJCA:

Rok urodzenia ………….………..... proszę wpisać o kogo chodzi ze strony ojca ..…………………....………..

| **ZDARZENIE** | | **(PRA)BABCIA** | | | **(PRA)DZIADEK** | | |
| --- | --- | --- | --- | --- | --- | --- | --- |
|  |  | **TAK** | **NIE** | **NIE WIEM** | **Tak** | **Nie** | **NIE WIEM** |
| **Czy podczas wojny Pani/Pana (pra)babcia/dziadek:** | | | | | | | |
| 1. | Straciła/stracił matkę? |  |  |  |  |  |  |
| 2. | Straciła/stracił ojca? |  |  |  |  |  |  |
| 3. | Stracił/a inną bliską osobę? |  |  |  |  |  |  |
| 4. | Brał/a udział w walce frontowej? |  |  |  |  |  |  |
| 5. | Był/a w partyzantce? |  |  |  |  |  |  |
| **ZDARZENIE** | | **(PRA)BABCIA** | | | **(PRA)DZIADEK** | | |
|  |  | **TAK** | **NIE** | **Nie** | **Tak** | **Nie** | **Nie** |

|  | |  |  | **wiem** |  |  | **wiem** |
| --- | --- | --- | --- | --- | --- | --- | --- |
| **Czy podczas wojny Pani/Pana (pra)babcia/dziadek:** | | | | | | | |
| 6. | Był/a ranny/a? |  |  |  |  |  |  |
| 7. | Zabił/a kogoś? |  |  |  |  |  |  |
| 8. | Był/a torturowana? |  |  |  |  |  |  |
| 9. | Był/a w hitlerowskim obozie koncentracyjnym? |  |  |  |  |  |  |
| 10. | Był/a w łagrze? |  |  |  |  |  |  |
| 11. | Był/była w getcie? |  |  |  |  |  |  |
| 12. | Była w Warszawie podczas  Powstania Warszawskiego? |  |  |  |  |  |  |
| 13. | Doświadczył/a gwałtu lub innej formy przemocy seksualnej? |  |  |  |  |  |  |
| 14. | Przeżył/a bombardowanie? |  |  |  |  |  |  |
| 15. | Musiał/a się ukrywać? |  |  |  |  |  |  |
| 16. | Ukrywał/a Żydów? |  |  |  |  |  |  |
| 17. | Był/a przymusowo wysiedlona na Syberię? |  |  |  |  |  |  |
| 18. | Był/a na robotach przymusowych  na terenie III Rzeszy? |  |  |  |  |  |  |
| 19. | Doświadczył/a zagrażającego życiu  mrozu? |  |  |  |  |  |  |
| 20. | Doświadczył/a zagrażającego życiu głodu? |  |  |  |  |  |  |
| 21. | Walczył/a w partyzantce lub w ruchu oporu (Armia Krajowa, Gwardia Ludowa, innych)? |  |  |  |  |  |  |
| 22. | Był/a poważnie chora? |  |  |  |  |  |  |
| **ZDARZENIE** | | **(PRA)BABCIA** | | | **(PRA)DZIADEK** | | |

|  | | **TAK** | **NIE** | **NIE WIEM** | **TAK** | **NIE** | **NIE WIEM** |
| --- | --- | --- | --- | --- | --- | --- | --- |
| **Czy podczas wojny Pani/Pana (pra)babcia/dziadek była/był świadkiem:** | | | | | | | |
| 23. | Przeżył/a fakt, że ktoś z jego/jej najbliższych był poddany  torturom, doznał gwałtu,  przemocy seksualnej lub został poważnie ranny? |  |  |  |  |  |  |
| 24. | Walki frontowej? |  |  |  |  |  |  |
| 25. | Postrzelenia kogoś? |  |  |  |  |  |  |
| 26. | Egzekucji, zabicia kogoś? |  |  |  |  |  |  |
| 27. | Gwałtu lub innej formy przemocy  seksualnej? |  |  |  |  |  |  |
| 28. | Ciężkiego pobicia kogoś? |  |  |  |  |  |  |
| 29. | Napaści lub prześladowania Żydów? |  |  |  |  |  |  |
