# Supplementary material for: Knowledge about traumatic World War II experiences among ancestors and subjective well-being of young adults: A person-centred perspective
Source: PLoS One. 2020 Aug 24;15(8):e0237859. doi: 10.1371/journal.pone.0237859 (PMC7446788; doi:10.1371/journal.pone.0237859)
Supplement: S2 File — (DOCX) [file pone.0237859.s002.docx]

**KNOWLEDGE ABOUT WORLD WAR II EVENTS: QUESTIONNAIRE**

Maja Lis-Turlejska, Anna Ćwiklińska, Piotr Halimoniuk

This questionnaire was created with the intention of establishing the knowledge regarding traumatic events experienced by your ancestors which occurred during the second world war. Depending on their age this may be your great grandparents or grandparents.

Below you will find two tables. Please read the questions on the left hand side and mark (X) in the rubic YES if you know that such an event occured – your great grandparent or grandparent talked to you about it or you found out about it in a different way. If such an event did not occur, please mark NO in the rubric. If you don't know anything regarding this topic, please mark DON'T KNOW in the rubric. If any of your ancestors was born after 1945, please omit the relevant columns. The first table regards the ancestors from your mother's side of the family. The second, your father's side of the family.

**(GREAT)GRANDFATHER/(GREAT)GRANDMOTHER FROM MOTHER'S SIDE:**

Date of birth................

Please fill in who you have chosen from your mother’s side of family……………..

| **EVENT** | | **(GREAT)GRANDMOTHER** | | | **(GREAT)GRANDFATHER** | | |
| --- | --- | --- | --- | --- | --- | --- | --- |
|  |  | **YES** | **NO** | **DON’T KNOW** | **YES** | **NO** | **DON’T KNOW** |
| **During the war did your (great) grandmother/grandfather:** | | | | | | | |
| 1. | Lose their mother? |  |  |  |  |  |  |
| 2. | Lose their father? |  |  |  |  |  |  |
| 3. | Lose another close person? |  |  |  |  |  |  |
| 4. | Fight on the front lines? |  |  |  |  |  |  |
| 5. | Partake in guerrilla warfare? |  |  |  |  |  |  |
| 6. | Get injured? |  |  |  |  |  |  |
| 7. | Kill someone? |  |  |  |  |  |  |
| 8. | Get tortured? |  |  |  |  |  |  |
| **EVENT** | | **(GREAT)GRANDMOTHER** | | | **(GREAT)GRANDFATHER** | | |
|  |  | **YES** | **NO** | **DON’T KNOW** | **YES** | **NO** | **DON’T KNOW** |
| **During the war was your (great) grandmother/grandfather:** | | | | | | | |
| 9. | In a Nazi concentration camp? |  |  |  |  |  |  |
| 10. | In a labour camp? |  |  |  |  |  |  |
| 11. | In a ghetto? |  |  |  |  |  |  |
| 12. | In Warsaw during the Warsaw Uprising? |  |  |  |  |  |  |
| 13. | Raped or did they experience another form of sexual assault? |  |  |  |  |  |  |
| 14. | Survive a bombing? |  |  |  |  |  |  |
| 15. | In need of hiding? |  |  |  |  |  |  |
| 16. | Hiding Jews? |  |  |  |  |  |  |
| 17. | Forcibly deported to Siberia? |  |  |  |  |  |  |
| 18. | On the territory of the Third Reich for forced labour? |  |  |  |  |  |  |
| 19. | Experiencing life threatening cold? |  |  |  |  |  |  |
| 20. | Experiencing life threatening hunger? |  |  |  |  |  |  |
| 21. | Fighting in guerrilla warfare or in a resistance movement (Home Army, Polish National Army etc.)? |  |  |  |  |  |  |
| 22. | Seriously ill? |  |  |  |  |  |  |
| 23. | Experiencing the fact that someone in their family was tortured, raped, sexually assaulted or seriously injured? |  |  |  |  |  |  |
| **EVENT** | | **(GREAT)GRANDMOTHER** | | | **(GREAT)GRANDFATHER** | | |
|  |  | **YES** | **NO** | **DON’T KNOW** | **YES** | **NO** | **DON’T KNOW** |
| **During the war was your (great) grandmother/grandfather a witness to:** | | | | | | | |
| 24. | Fighting on the front lines? |  |  |  |  |  |  |
| 25. | Someone being shot? |  |  |  |  |  |  |
| 26. | Execution, murder? |  |  |  |  |  |  |
| 27. | Rape or other form of sexual assault? |  |  |  |  |  |  |
| 28. | Severe physical assault? |  |  |  |  |  |  |
| 29. | Assault or persecution of Jews? |  |  |  |  |  |  |

**(GREAT)GRANDFATHER/(GREAT)GRANDMOTHER FROM FATHER'S SIDE:**

Date of birth................

Please fill in who you have chosen from your mother’s side of family……………..

| **EVENT** | | **(GREAT)GRANDMOTHER** | | | **(GREAT)GRANDFATHER** | | |
| --- | --- | --- | --- | --- | --- | --- | --- |
|  |  | **YES** | **NO** | **DON’T KNOW** | **YES** | **NO** | **DON’T KNOW** |
| **During the war did your (great) grandmother/grandfather:** | | | | | | | |
| 1. | Lose their mother? |  |  |  |  |  |  |
| 2. | Lose their father? |  |  |  |  |  |  |
| 3. | Lose another close person? |  |  |  |  |  |  |
| 4. | Fight on the front lines? |  |  |  |  |  |  |
| 5. | Partake in guerrilla warfare? |  |  |  |  |  |  |
| 6. | Get injured? |  |  |  |  |  |  |
| 7. | Kill someone? |  |  |  |  |  |  |
| 8. | Get tortured? |  |  |  |  |  |  |
| **EVENT** | | **(GREAT)GRANDMOTHER** | | | **(GREAT)GRANDFATHER** | | |
|  |  | **YES** | **NO** | **DON’T KNOW** | **YES** | **NO** | **DON’T KNOW** |
| **During the war was your (great) grandmother/grandfather:** | | | | | | | |
| 9. | In a Nazi concentration camp? |  |  |  |  |  |  |
| 10. | In a labour camp? |  |  |  |  |  |  |
| 11. | In a ghetto? |  |  |  |  |  |  |
| 12. | In Warsaw during the Warsaw Uprising? |  |  |  |  |  |  |
| 13. | Raped or did they experience another form of sexual assault? |  |  |  |  |  |  |
| 14. | Survive a bombing? |  |  |  |  |  |  |
| 15. | In need of hiding? |  |  |  |  |  |  |
| 16. | Hiding Jews? |  |  |  |  |  |  |
| 17. | Forcibly deported to Siberia? |  |  |  |  |  |  |
| 18. | On the territory of the Third Reich for forced labour? |  |  |  |  |  |  |
| 19. | Experiencing life threatening cold? |  |  |  |  |  |  |
| 20. | Experiencing life threatening hunger? |  |  |  |  |  |  |
| 21. | Fighting in guerrilla warfare or in a resistance movement (Home Army, Polish National Army etc.)? |  |  |  |  |  |  |
| 22. | Seriously ill? |  |  |  |  |  |  |
| 23. | Experiencing the fact that someone in their family was tortured, raped, sexually assaulted or seriously injured? |  |  |  |  |  |  |
| **EVENT** | | **(GREAT)GRANDMOTHER** | | | **(GREAT)GRANDFATHER** | | |
|  |  | **YES** | **NO** | **DON’T KNOW** | **YES** | **NO** | **DON’T KNOW** |
| **During the war was your (great) grandmother/grandfather a witness to:** | | | | | | | |
| 24. | Fighting on the front lines? |  |  |  |  |  |  |
| 25. | Someone being shot? |  |  |  |  |  |  |
| 26. | Execution, murder? |  |  |  |  |  |  |
| 27. | Rape or other form of sexual assault? |  |  |  |  |  |  |
| 28. | Severe physical assault? |  |  |  |  |  |  |
| 29. | Assault or persecution of Jews? |  |  |  |  |  |  |
