# Supplementary material for: Knowledge about traumatic World War II experiences among ancestors and subjective well-being of young adults: A person-centred perspective
Source: PLoS One. 2020 Aug 24;15(8):e0237859. doi: 10.1371/journal.pone.0237859 (PMC7446788; doi:10.1371/journal.pone.0237859)
Supplement: Attachment — Submitted filename: Response to reviewers_vol2.docx [file pone.0237859.s005.docx]

**Dear Editor, Dear Reviewers,**

**thank you very much for another suggestions and remarks concerning our article titled “Knowledge about Traumatic World War II Experiences among Ancestors and Subjective Well-being of Young Adults: A Person-Centred Perspective”, which we would like to publish in *PLOS One*. We referred to all reviewers’ remarks. Below we cite every remark and comment of the reviewers and provide the answers to them in parentheses. All the changes in the revised text are marked with red font.**

**Reviewers' comments:**

Reviewer's Responses to Questions

Reviewer #1: The authors have responded to most of my comments yet several issues remained unresolved.

**[Thank you very much for kind words. We tried a lot to incorporate all your remarks; however, we see that not all were incorporated completely properly.]**

(1) Regarding the claim that second and third generations reported higher levels of physical health problems like cancer, heart disease, or chronic pain: In their response the authors mention neuroendocrine and epigenetic works. Still it seems like they stretched the conclusions of these studies in their claim for higher physical morbidity. I also don't think Baranovsky or Sigal & Weinfeld provided empirical evidence for higher physical morbidity in these groups. Therefore I ask the authors

to rephrase the sentence or alternatively provide empirical support for their claim.

**[Thank you very much for this remark. It is very important suggestion. As we wrote in the previous response to your comments, there are many hypotheses on how parental trauma may affect next generation mental and physical health. Due to the cross-sectional nature of this study and related limitations, we did not want to elaborate on this issue in details to avoid some speculative remarks taking into an account our study design and assessed variables. And in the introduction we only mentioned that such theories exist. However, at this time, in the second revision of our study we eliminated from the discussion sections all mentioning about neuroendocrine and epigenetic mechanisms to comment our findings. We also eliminated Baranovsky and Sigal & Weinfeld references when writing about higher physical morbidity in these groups. Once again thank you for this remark.]**

(2) Shrira is misspelled as Shira twice; please correct.

**[Thank you very much for this remark – we corrected this typo throughout the whole manuscript.]**

**To sum up, I would like to thank again Editor and Reviewers for their time and effort. I found all the comments very useful and I believe that they helped me to improve the manuscript quality. I deeply appreciate a chance you gave me to revise and submit it to be considered for publication in *PLOS One.***
